# Removal of Multiple Ingested Magnets Through Laparoscopic Appendectomy in an Adolescent: A Report of Two Cases

**DOI:** 10.7759/cureus.58825

**Published:** 2024-04-23

**Authors:** Kaja Gizewska-Kacprzak, Karol Nicieja, Bartosz Gajek, Lidia Babiak-Choroszczak

**Affiliations:** 1 Department of Pediatric and Oncological Surgery, Urology and Hand Surgery, Pomeranian Medical University in Szczecin, Szczecin, POL

**Keywords:** acute abdomen, appendectomy, laparoscopy, children, magnet ingestion

## Abstract

Magnet ingestion can lead to serious health issues, including inflammation, gastrointestinal tract perforation, and even life-threatening complications. Despite legislative actions and numerous reports on the dangers of magnet ingestion in children, it remains a significant public health concern. Physicians must remain vigilant in cases of acute abdomen with ambiguous symptoms or unclear history in young patients. Prompt diagnosis and surgical intervention in case of multiple magnet swallowing are crucial to prevent complications. We present two cases of successful removal of ingested magnetic spheres through laparoscopic appendectomy in adolescents. This study aimed to highlight the technical aspects of the procedure to share the benefits of minimally invasive surgery (MIS) in the management of magnetic foreign bodies (FBs) located in the appendix or cecum.

## Introduction

Single magnet ingestion is not associated with an increased risk of morbidity. However, it is hazardous if not caught early, involves multiple magnets, or if a battery is co-ingested [1-5]. Even two magnets located at different levels of the intestine can merge through bowel loops and cause various life-threatening complications, including numerous perforations of the gastrointestinal tract (GI), ischemia or necrosis of the intestines, the development of fistulas, peritonitis, and obstruction [4,6-9]. Therefore, magnetic sphere ingestion can pose a significant health risk in a pediatric population. Children under six years of age are at highest risk, and most of them are boys [10]. There is no typical presentation in magnet ingestion, so a child's age, reluctance, or inability to describe circumstances can delay diagnosis [4-8]. The issue led to the implementation of legislative regulations at the federal level in 2012 to prohibit magnetic toy distribution in the United States of America [1]. Fortunately, most cases can be managed conservatively or with endoscopic removal of the foreign body. However, ingesting multiple magnets usually requires surgical intervention [1]. Sixty percent of patients undergo explorative laparotomy, and only 20% of surgeries are entirely laparoscopic. Magnets are removed with an appendectomy in 3% of cases [7]. In the era of widespread minimally invasive surgery (MIS) in children, we present two cases of successful laparoscopic management of multiple magnet ingestion. Both cases are unusual due to the patient's age, an extended period of conservative treatment, and resolution via laparoscopic appendectomy.

## Case presentation

Case 1

**Figure 1:**
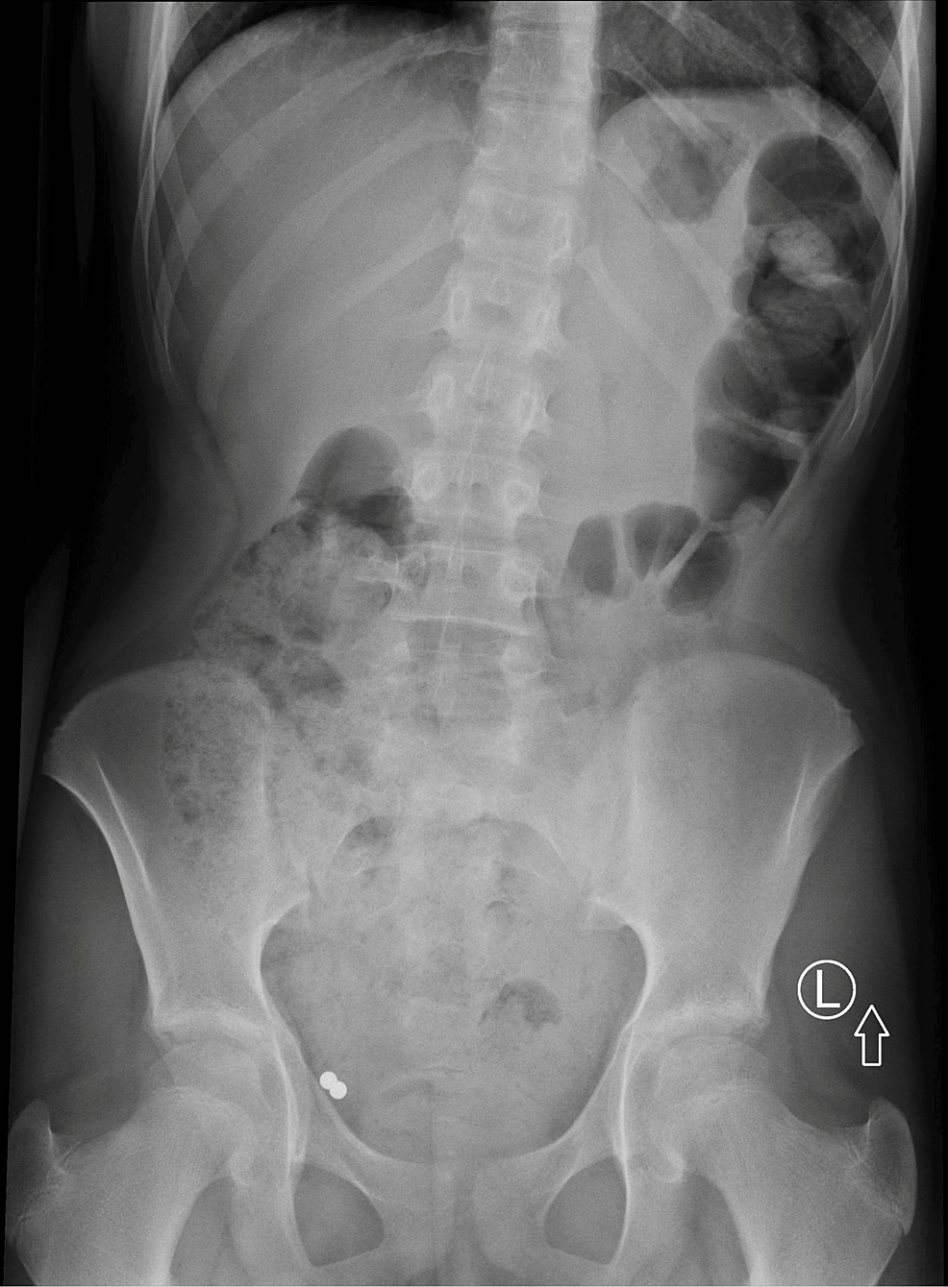
Abdominal X-ray showing two magnetic balls in the right lower quadrant.

**Figure 2:**
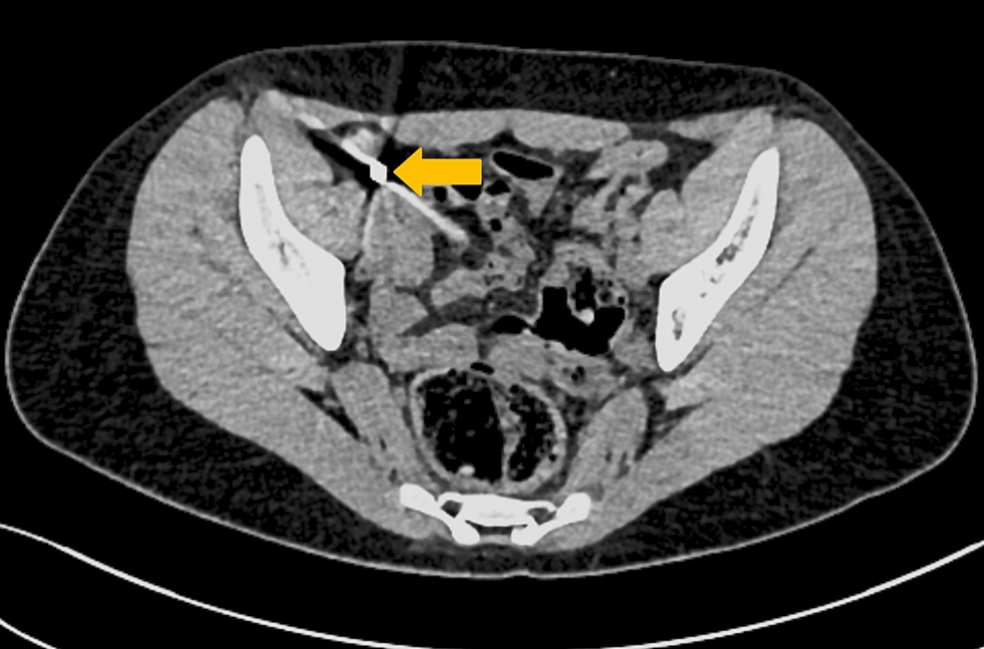
An abdominal low-dose computed tomography showing two magnetic balls in the lumen of the appendix.

A 10-year-old girl was admitted to the pediatric surgery department after swallowing two magnets, each 5 mm in diameter. The patient admitted to ingesting magnetic spheres during an evening play and was rushed to the pediatric emergency department at the time of the incident. Surprisingly, the gastroscopy was postponed for several hours, and objects passed further into the GI tract. Consequently, she was treated for 18 days conservatively in a pediatric gastroenterology ward, which included five X-rays and multiple enemas. There was no progression in the movement of the foreign bodies (FBs) in the right lower quadrant in subsequent X-rays. Afterward, an attempt was made to identify and extract the magnetic spheres through a colonoscopy. The objects were not found in the cecum, with a suspicion of passing into the lumen of the appendix. The child was referred to the pediatric surgery department at a different hospital. On admission, she was in good general condition and showed no symptoms. An abdominal X-ray confirmed the presence of the two magnetic spheres (Figure 1). No FBs were seen on the abdominal ultrasound. As the history was over two weeks with a risk of extra lumen migration of the FBs, a low-dose contrast-free abdominal computed tomography (CT) was performed to exclude further damage to the bowel or the need for explorative laparotomy. Two metal foreign bodies were visualized in the lumen of the appendix without any signs of perforation (Figure 2 and Video 1).

**Video 1:** Video of an abdominal computed tomography showing two magnetic balls in the lumen of the appendix.

**Figure 3:**
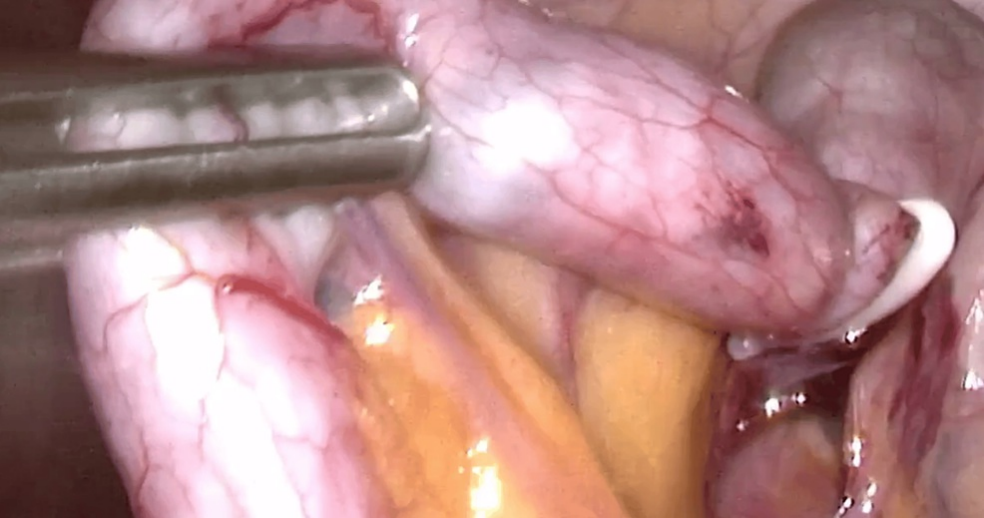
Intraoperative laparoscopic picture: magnetic spheres were visible through the wall of the appendix.

The girl was qualified for a laparoscopic appendectomy with planned plastic single-use trocars to avoid the movement of the magnets. The laparoscopic approach allowed visualization of the appendix in an anterior position at the level of the iliac vessels. Magnets were seen in the lumen of the appendix and immediately moved toward the metal laparoscopic instruments and stuck to their surface. The base of the appendix was closed after local blunt dissection with 3 polymer clips so that the magnets could not move back to the cecum. Coagulation was not used to avoid any thermal interaction with metal objects. The mesoappendix was clipped. Both the appendix and mesoappendix were cut off, and the specimen containing two magnets in the lumen was evacuated through the umbilical trocar (Figure 3 and Video 2). Histopathology confirmed areas of inflammation. The child was discharged on the fourth day, and long-term follow-up showed no complications.

**Video 2:** Laparoscopic appendectomy with removal of two magnetic spheres in the lumen of the appendix.

Case 2

**Figure 4:**
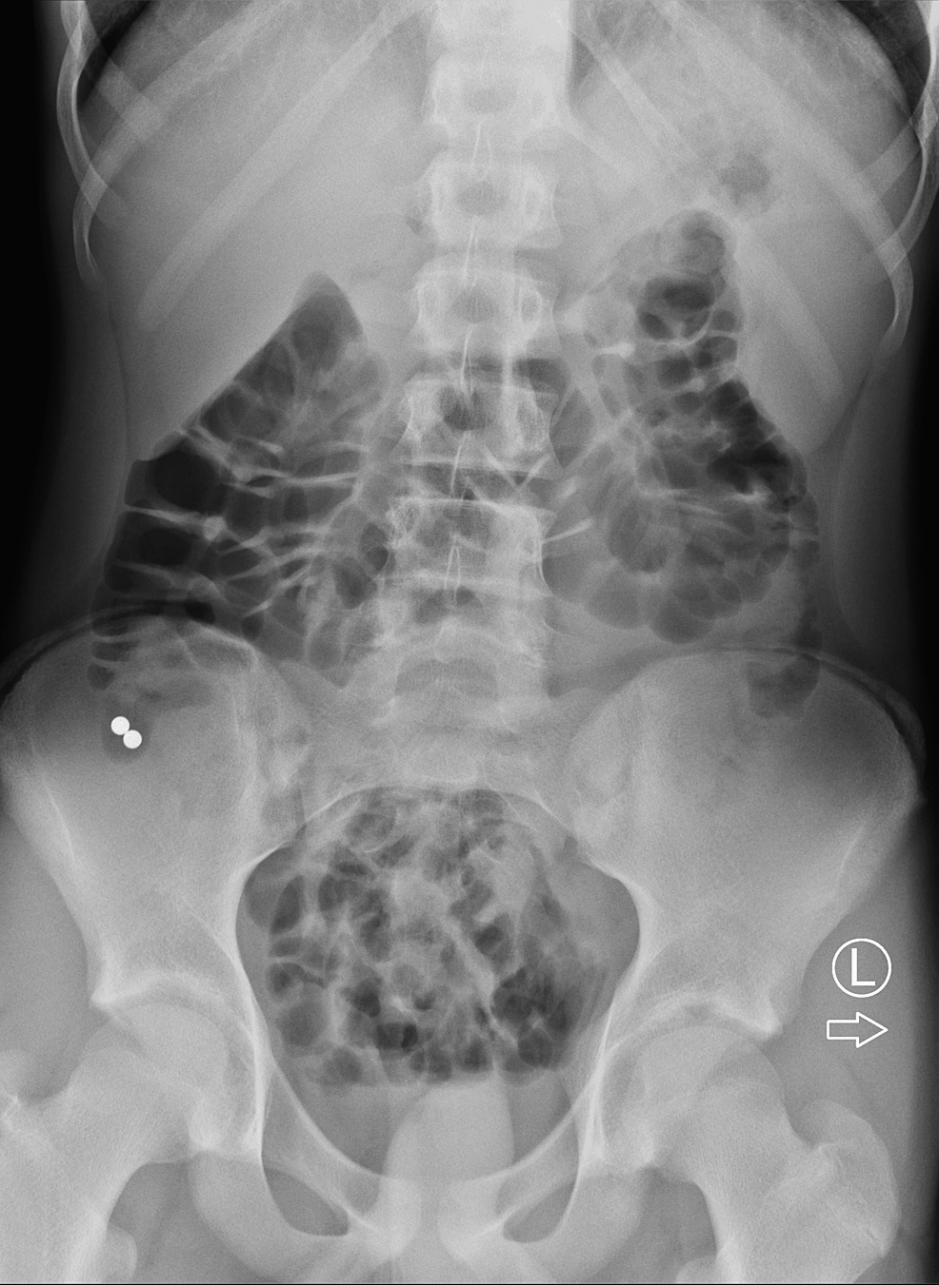
Abdominal X-ray showing two magnetic balls in the cecum.

A 13-and-a-half-year-old boy swallowed two magnetic spheres and was admitted to the pediatric gastroenterology department. The boy could not explain the circumstances of the incident. Most probably, he was holding them in his mouth while constructing and did it unintentionally. He was in good general condition on admission, with no signs of acute abdomen. As the objects passed the stomach, conservative management was implemented. With no progress in the passing of magnets after seven days, an ileocolonoscopy was performed with no success. An abdominal X-ray confirmed the presence of the two magnets in the lower-right quadrant of the abdomen (Figure 4).

FBs were not visualized on the abdominal ultrasound. He was referred to the pediatric surgery ward. Additional abdominal CT scans showed two metal objects in the cecum, with no signs of perforation (Video 3).

**Video 3:** Abdominal low-dose computed tomography showing two magnetic balls in the cecum.

**Figure 5:**
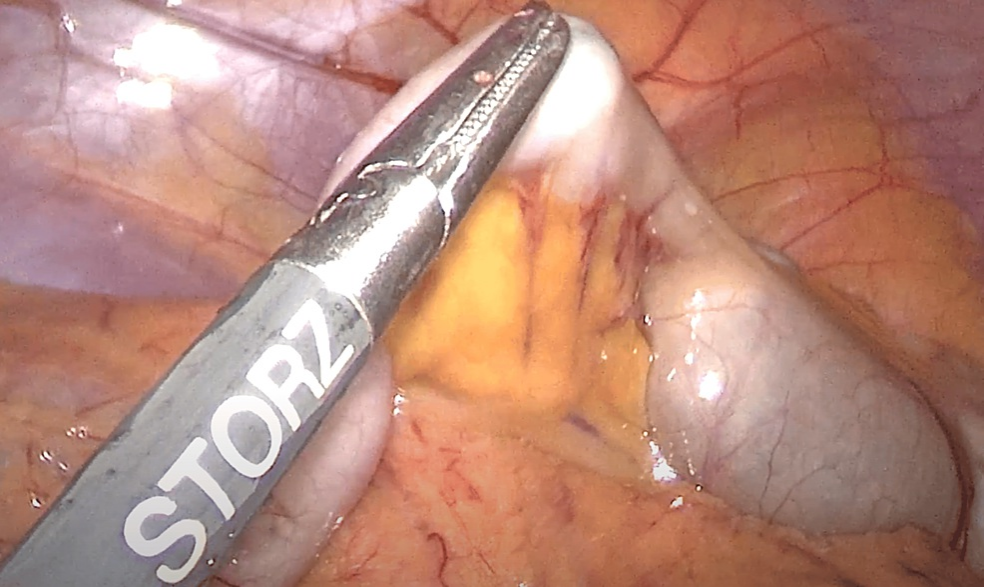
Magnetic spheres inside the appendix stuck to the grasper and pulled distally.

**Figure 6:**
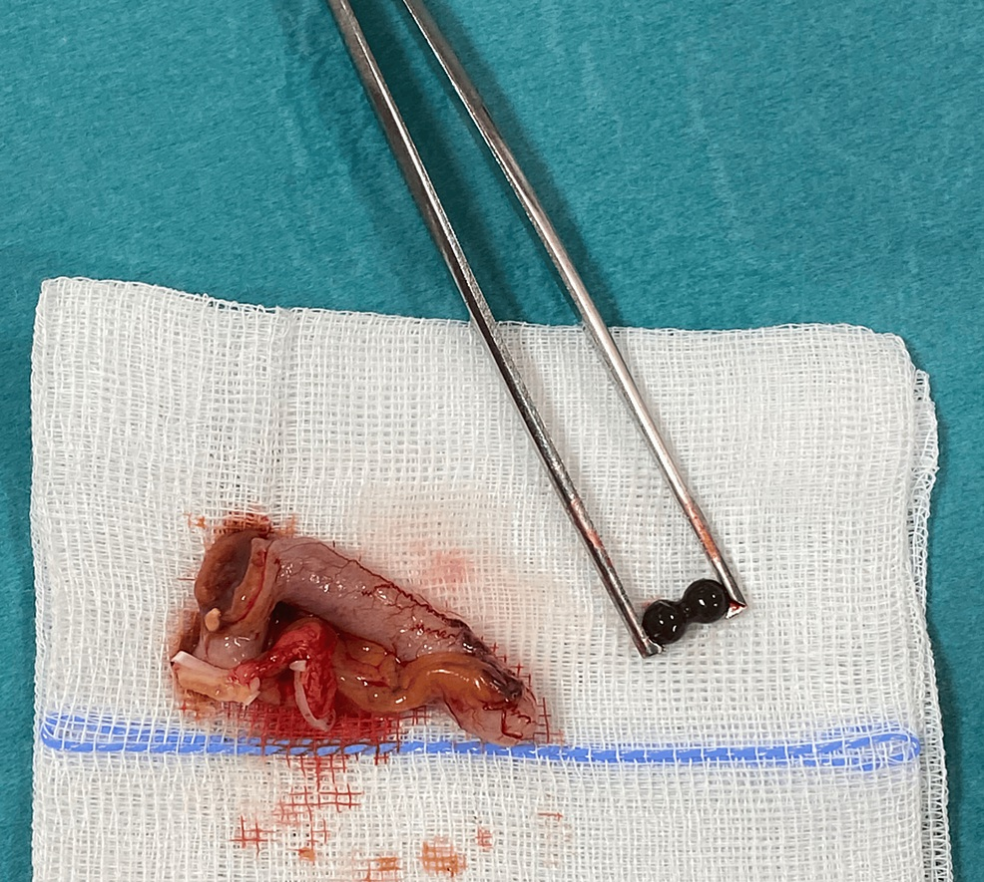
Magnetic balls excised from the appendix postoperatively.

On the eighth day of hospitalization, a laparoscopy was performed. The appendectomy was carried out in the same setting as described in the previous case. The appendix was also positioned anteriorly. However, the magnets were seen at the basis of the appendix and pulled distally by the mutual attraction between the magnetic spheres and laparoscopic instruments further into the lumen of the appendix at the beginning of the procedure (Figures 5, 6, and Video 4). Pathology confirmed purulent appendicitis. The child was discharged four days later, and long-term follow-up was uneventful.

**Video 4:** Laparoscopic appendectomy with removal of the two magnetic balls in the appendix.

## Discussion

Magnetic sphere ingestion is a well-described major health hazard in children. With an incidence of over 1000 cases per year in the United States alone, pediatric surgeons, gastroenterologists, and physicians working in emergency departments must be aware of the issue and available therapeutic options. In the United States, multiple legislative procedures were undertaken in the past to limit magnet accessibility for children, with an unfortunate pushback from the industry. An almost 4.5 times increase in ingestion incidents was reported in 2018 after magnets were allowed back on the market [10].

Two age peaks are observed at two to four years and eight to 10 years, with rare magnet ingestion in children over 13 years [1,2]. Therefore, our two cases of adolescent patients, including the second case of a 13-and-a-half-year-old, are unusual. Interestingly, one-fourth of incidents in older children involve using magnetic spheres as fake piercings or are associated with comorbid psychiatric disorders [11]. Our patient stories prove that even though magnet ingestion is rare in older children, it cannot be ruled out based on age alone. A thorough medical history should be taken in all cases of suspected magnetic FBs ingestion.

Notably, the period between the ingestion and the onset of symptoms can vary significantly, lasting up to 40 days [6]. The majority of patients are asymptotic but may also present with nonspecific symptoms like abdominal pain or distension, vomiting, loss of appetite, and fever [6,7]. Unfortunately, in some cases, the progression is rapid and fatal. Olczak and Skrzypek presented a tragic lethal case of an eight-year-old boy who passed after cardiac arrest due to mesenteric volvulus of the enteric loop caused by only two magnetic spheres that twisted the bowel loop and vessels [4].

The risk of complications increases with the number of swallowed magnets [1]. Thus, assessment of the number of FBs is crucial in the management of magnet ingestion. Yet, it is difficult to estimate when the patient's medical history is unclear. It is especially prominent in the case of very young patients, as almost 3/4 of incidences are unwitnessed [7]. Additionally, their diameter can also impact the prognosis, with magnets smaller than 5 millimeters being associated with higher morbidity [1]. Regardless of circumstances, a chest and abdominal X-ray is a gold standard used to confirm the presence of metallic FBs in the GI [12]. A series of X-rays are used in the conservative treatment of multiple magnet ingestion to track their passage progress [3,12]. However, it is noteworthy that it does not allow distinguishing metallic and magnetic FBs. A low-dose CT scan can better assess the magnet location or potential complications [13]. As magnets only need hours to cause ulceration, extended conservative treatment poses a risk of complications [14]. Since both patients had histories of prolonged observation of not moving magnets, low-dose CTs were performed to exclude extra lumen location of objects or further damage to surrounding organs. Ultrasound is a safe and accurate noninvasive modality used to determine the magnet location [6]. However, those examinations were inconclusive in our cases. Fluoroscopy can help locate metal FBs during endoscopy or surgery [13,15]. Magnetic resonance imaging (MRI) is strictly contradicted in all cases of metallic FBs ingestion as it may cause catastrophic and life-threatening complications. Bailey et al. reported multiple perforations after a neck MRI in a five-year-old boy who had ingested 11 magnets before the examination. The child complained of acute neck pain, and there was no suspicion of the magnetic spheres ingestion. After explorative laparotomy, the history was updated with a confirmation of an unattended play with magnets before admission [16].

The North American Society of Pediatric Gastroenterology Hepatology and Nutrition (NASPGHAM) revised comprehensive guidelines for the management of magnet ingestion in children in 2015 [3]. Subsequently, in 2023, the European Society for Pediatric Gastroenterology Hepatology and Nutrition (ESPGHAN) published its position paper with up-to-date clinical guidelines [17]. However, signs of aspiration or esophageal obstruction indicate an immediate need for intervention, we know from the literature that single magnet ingestion is associated with no risk of morbidity. A watch-and-wait approach can be implemented with at-home observation and stool inspection to confirm FB passage in case of a confirmed single magnet below the lower esophageal sphincter [1]. It is imperative to remember that, in some cases, imaging techniques may lack sensitivity. Several cases of multiple magnet ingestion simulating ingestion of a single foreign body have been reported [18,19]. Conservative treatment should be carefully considered in all uncertain cases of magnetic FBs ingestion.

Endoscopic removal of FBs through gastroscopy is the first line of treatment for multiple magnet ingestion and significantly decreases the risk of complications [3,17]. However, delayed diagnosis makes it hard to achieve, as in our case. If they have passed the stomach, the patient has to be monitored for their progression and informed about the possible need for surgery. In 2018, Sola et al. proposed possible indications for surgical intervention. Physicians should consider surgery when faced with signs of perforation, symptomatic patients with magnets beyond the stomach, or no progression on abdominal X-ray within 48 hours [20]. Therefore, the initial prolonged observation of our two patients risked complications and unnecessary repetition of X-rays.

Intervals between the ingestion of each magnet may result in locating them in different segments of the intestine. As the ingestion regards high-power magnets, their attractive forces can lead them to merge even through several bowel loops, causing perforations, necrosis, or fistulas [6]. Hussain et al. observed gastric ulceration during the endoscopic removal of multiple magnets less than 8 hours after swallowing, emphasizing the harmful effects on the upper GI tract [14]. If the patient displays symptoms of an acute abdomen or magnets are visualized at various levels of the intestines, the decision to operate should not be delayed [6]. The tragic consequences of intermittent swallowing of multiple magnets seem to be reserved for numerous objects. Ingesting even two magnets at the same time is already associated with an increased risk of complications. However, it occurs in only about 7.5% of cases [1]. Naji et al. suggested that the magnets might get separated by the peristaltic movement and detach from one another to connect in other positions that risk perforation [21]. This further emphasizes the dangers of prolonged observation. With the extreme example of lethal volvulus after ingestion of two magnetic spheres, as mentioned before, the risk of surgery significantly outweighed possible complications. Furthermore, FBs located in the lumen of the appendix are unlikely to be passed yet can cause inflammation or necrosis, even after several years [15]. Therefore, in both of the presented children, our team consult resulted in surgical exploration after radiologically supported preoperative planning of a laparoscopic approach, as the conservative approach was already dangerously prolonged.

Hayward and Saxena analyzed 136 cases of multiple magnet ingestion in children, comparing surgical approaches. They showed a significant reduction in the number and severity of complications and reduced length of stay in cases treated with MIS [7]. Magnetic FB removal with appendectomy has been previously well described in the literature. Sun et al. proposed the Magnet Extraction Through Appendectomy Laparoscopically (METAL) technique, which involves milking the magnets to the appendix [22]. With a low risk of the procedure and possible complications in mind, laparoscopic appendectomy should be considered as the treatment of choice in case of magnets located in the colon or the lumen of the appendix [7,21,23]. However, when inexperienced, faced with multiple perforations, difficult anatomical conditions, or a need for bowel resection, laparotomy should be considered [7].

In our cases, we were able to repeat and record almost identical approaches. In both procedures, we restrained from using coagulation to prevent appendix perforation by warmed metallic spheres. As the magnets may stick to metallic instruments, the lumen of the appendix was closed with plastic clips as the first step of the procedure. As the authors of the abovementioned reports have not shared those details or video material, we hope that the material provided will be a useful source for fellow surgeons and may provide practical guidance in similar cases as the promotion of MIS.

Although the treatment courses of our patients were uneventful, it is worth emphasizing the importance of prevention. A social campaign should be addressed to parents to inform them about the potential dangers of magnetic object ingestion, as a significant portion of magnetic FB ingestion happens at home [1,2]. Easy-to-swallow FBs should be kept away from young children still exploring the world around them, as most ingested magnets were intended for adults [2]. Further legislative actions should be undertaken to ban or impose stricter regulations on magnetic sphere sales [17].

## Conclusions

Magnet ingestion may affect children of all ages. Prompt diagnosis and eventual surgical intervention in case of multiple magnets ingestion are crucial to prevent complications. Prolonged observation is unjustified. Removal of magnetic FBs through laparoscopic appendectomy is a valid and safe management option in selected cases, especially if they are already located in the appendix. Practical tips can adapt the technique to the presence of magnetic objects. Above all, the general public should be educated about the danger of magnets ingestion.
